# Investigation on sleep-related cognition of Chinese health care workers during the first wave of COVID-19 pandemic

**DOI:** 10.3389/fpsyt.2023.1019837

**Published:** 2023-03-13

**Authors:** Wei Wang, Xincan Ji, Hao-Yang Guo, Mengjun Tao, Lairun Jin, Miao Chen, Hui Yuan, Hui Peng

**Affiliations:** ^1^School of Public Health, Wannan Medical College, Wuhu, Anhui, China; ^2^Health Management Center, The First Affiliated Hospital of Wanan Medical Collegue, Wuhu, Anhui, China; ^3^School of Public Health, Southeast University, Nanjin, Jiangsu, China; ^4^Department of Science and Technology Administration, The First Affiliated Hospital of Wanan Medical Collegue, Wuhu, Anhui, China

**Keywords:** sleep-related cognition, dysfunctional beliefs about sleep, sleep quality, health care workers, COVID-19

## Abstract

**Background:**

The COVID pandemic has brought tremendous negative effects on the mental health of health care workers, such as anxiety, depression, and sleep disorders. We conducted this study to evaluate the sleep-related cognition of Chinese health care workers (HCWs) during the first wave of COVID-19 pandemic and analyze its association with sleep quality, so as to provide scientific reference for improving sleep of HCWs.

**Patients and methods:**

A total of 404 HCWs from Yijishan Hospital of Wuhu City, China were enrolled in the study, selected by randomized cluster sampling in May 2020. We made a questionnaire to collect the general demographic information of the participants. The Pittsburgh Sleep Quality Index (PSQI) and a brief version of Dysfunctional Beliefs and Attitudes about Sleep Scale (DBAS-16) were used to measure sleep quality and sleep-related cognition, respectively.

**Results:**

The results showed that 312 HCWs (77.2%) had false beliefs and attitudes about sleep, while only 92 HCWs (22.8%) had correct beliefs about sleep. In addition, we found that those HCWs who were older, married, with a bachelor’s degree or higher, nurses, more daily working hours (> 8 h) and monthly night shifts (≥ 5 times), had higher DBAS-16 scores (all *p* < 0.05). However, we did not find significant differences between men and women in DBAS-16 scores. According to the definition of PSQI, a total of 1/4 of the HCWs are poor sleepers and their DBAS-16 score was higher than good sleepers (*t* = 7.622, *p* < 0.001). In the end, we confirmed a positive correlation between sleep cognition and sleep quality (*r* = 0.392, *p* < 0.01).

**Conclusion:**

Our study revealed false beliefs and attitudes about sleep were prevalent among HCWs during the first wave of COVID-19 pandemic, and these false beliefs about sleep were closely correlated to sleep quality. We recommend fighting against these false beliefs about sleep.

## 1. Introduction

Sleep is an essential physiological need for human beings, whose basic function is to relieve fatigue and restore energy. In addition, sleep plays a very important role in growth and development, maintenance of mental health and cardiovascular metabolism (1–3). Adequate sleep helps flush out the neurotoxic waste that builds up during waking hours from the brain (4). Lack of sleep often leads to fatigue, poor concentration, slow reactions and impaired judgment, which increase the risk of traffic accidents, industrial accidents, medical errors, and reduced productivity (5, 6). Moreover, long-term insomnia can cause anxiety, depression, and affect immune function, memory and cognitive function (7–9).

Many studies have shown that sleep disorders are related to sleep-related cognition (10–12), but the relevant mechanism is not yet clear. Harvey AG proposed a cognitive model of sleep disorders, showing that thinking activities can trigger autonomous arousal and emotional distress and excessive worry about sleep is a manifestation of dysfunctional sleep beliefs (13). Previous studies have shown that patients with mental diseases and sleep disorders are prone to early awakening and difficulty falling asleep (14, 15). They had wrong sleep beliefs and attitudes, such as paying more attention to the consequences caused by insomnia and worrying too much about sleep. And because they worry about sleep excessively and exaggerate its consequences, their insomnia symptoms may be sustained and developed. Through the intervention of distorted sleep beliefs in insomnia patients, sleep quality, mental and physical disorders can be effectively improved (16).

Because of the particularity of work, HCWs often have long working hours and frequent shift work (17). In their daily work, they also face complex clinical situations, high workload, as well as some emotional patients and family members of violence (18), they are burdened with great pressure. As a result, sleep deprivation is a common problem among medical professionals, and this can have potentially adverse effects on them personally and the patients they treat (19). In particular, during the COVID-19 pandemic, the rising number of novel coronavirus diagnoses has put an increasing burden on health care systems around the world, and HCWS easily become direct victims. Studies have shown that HCWS are one of the most vulnerable groups in COVID-19 outbreak, their mental health symptoms are worse than those of the general population (20, 21). Besides, HCWs are responsible for nucleic acid testing, managing patients and dealing with emergencies in their daily work, so they usually need to extend their working hours, long-term exposure to patients can increase the risk of infection. Anxiety, depression and stress/post-traumatic stress disorder the most prevailing COVID-19 pandemic-related mental health problems affecting HCWs, other mental health problems include burnout, fear of infection, phobias, somatic symptoms and substance abuse (6). During COVID-19, the world witnessed the vital role of HCWs. HCWs around the world, not just in China, are making enormous sacrifices. They are working day and night on the frontlines of the battle, regardless of personal safety, worrying about their families, and facing serious shortages of manpower and protective equipment. Many of them were also infected, and some even lost their lives. HCWs have made an outstanding contribution to the fight against COVID-19, but their sleep deprivation is often overlooked, with many complaining of difficulty getting good sleep. Even after leaving the COVID-19 ward, some of the frontline HCWs still had sleep disorders (22). Although an increasing number of studies have found an important role for cognitive factors in sleep onset and maintenance, few have been based on a large sample and variable combination among HCWs. Therefore, we hope to study the effect of cognitive factors during this major epidemic and to see whether the misperception of sleep is related to sleep disorders, to provide some reference for improving the poor sleep condition of HCWs.

## 2. Materials and methods

### 2.1. Patients

A descriptive cross-sectional study was conducted at Yijishan Hospital Affiliated to Wannan Medical College in Wuhu City, Anhui Province, China in May 2020. In this study, a total of 404 HCWs were selected by randomized cluster sampling and they were asked to fill out the study questionnaire. The exclusion criteria used in this study included individuals who submitted ineligible questionnaire or blank questionnaire, those who filled out the questionnaire wrongly, and those with inaccurate or incomplete demographic information needed for data analysis. These HCWs include clinicians, nurses, medical technicians, administrative staff and so on.

### 2.2. Methods

#### 2.2.1. General demographic information

General demographic information for the study participants included age, sex (male/female), marital status, education level, occupation type, number of night shifts (per month), and average hours worked (per day).

#### 2.2.2. Dysfunctional beliefs and attitudes about sleep scale (DBAS-16)

DBAS is widely used to measure and assess an individual’s views and attitudes toward sleep. The original 30-item scale was shortened to a 16-item, our study uses a scaled-down version of DBAS (23). DBAS-16 reflected participants’ beliefs and attitudes toward sleep in four different domains: (a) the consequences of insomnia (items 5, 7, 9, 12, and 16), (b) worry/helplessness about sleep (items 3, 4, 8, 10, 11, and 14), (c) Sleep expectations (items 1 and 2), and (d) Drugs (items 6, 13, and 15). Participants were asked to answer all 16 questions and circled a number ranging from 0 (strongly disagree) to 10 (strongly agree). The score of sub-scale can be computed by adding the sum of scores for the items and divided by the number of items making up each subscale. The total score of DBAS-16 is calculated by adding all items and dividing by 16. The higher the DBAS-16 score, the more distorted the sleep beliefs and attitudes. Finally, we considered the total score over/equal to 4 as false sleep beliefs, and less than 4 as right sleep beliefs according to a recent research (24).

#### 2.2.3. The Pittsburgh sleep quality index

PSQI is commonly used to assess of sleep quality in patients with sleep disorders, mental disorders and general population (25). It is a self-reported questionnaire with nine questions (19 items in total) that reflect the subjects’ sleep quality over the past few months. The test was divided into seven sub-scales: sleep quality (item 6), sleep duration (item 4), sleep latency (item 2 and 5a), sleep efficiency (item 1, 3, and 4), sleep disorder (item 5b-5j), use of sleep medication (item 7) and daytime dysfunction (item 8 and 9). Each subscale is scored on a scale of 0–3, with an overall score of 0–21. The researchers found that PSQI score = 7 was used for the cut-off point had high sensitivity and specificity, which was suitable for Chinese people (26). Therefore, we defined that the total score of PSQI for good sleepers was lower than 7, while the total score of PSQI for people with poor sleep quality was 7 or above. The lower the PSQI score, the better the sleep quality.

### 2.3. Statistical analysis

Epidata 3.0 software was used to enter data. The descriptive statistics approach was used to describe the demographic variables such as age, gender, marital status, education level, and profession. The *t*-test and one-way analysis of variance (ANOVA) were used for comparison among different demographic groups. The Student–Newman–Keuls (SNK) was used for *post hoc* analyzes. A two-tailed *value of p* < 0.05 was considered to be statistically significant. The statistical analysis of the data was performed using the statistical software SPSS 18.0.

### 2.4. Ethics approval

All participants were informed of the research intention and signed an informed consent form and volunteered to participate in the study. The study was approved by the Ethics Committee of Wannan Medical College.

## 3. Results

### 3.1. Demographic characteristics of the sample

A total of 450 questionnaires were distributed, while 404 valid questionnaires were collected after some blank or invalid questionnaires were excluded. The mean age of the participants was 25.10 ± 6.32 years, and there were 182 males (45.0%) and 222 females (55.0%). As defined by the PSQI score, 25% of the participants were poor sleepers. The average DBAS-16 total score of the participants was 5.11 ± 1.65, among which 312 (77.2%) had false beliefs about sleep, while only 92 medical workers (22.8%) had correct sleep beliefs about sleep. The demographic information of participants was shown in Table 1.

**Table 1:** Demographic characteristics of the study sample (*N* = 404).

Variables	Number (%)
Gender	
Male	182 (45.0)
Female	222 (55.0)
Age (years, Mean ± SD)	25.10 ± 6.32
Marital status	
Unmarried / Divorced / Widowed	322 (79.7)
Married	82 (20.3)
Education	
Below bachelor	53 (13.1)
Bachelor	280 (69.3)
Master / Doctor	71 (17.6)
Profession	
Clinician	184 (45.5)
Nurse	29 (7.2)
Medical technician	108 (26.7)
Administrative staff	21 (5.2)
Other	62 (15.4)
PSQI	
<7	303 (75.0)
≥7	101 (25.0)
DBAS-16 (Mean ± SD)	5.11 ± 1.65
<4	92 (22.8)
≥4	312 (77.2)

Abbreviation: SD, Standard deviation; DBAS-16, Dysfunctional Beliefs and Attitudes about Sleep Scale; PSQI, Pittsburgh Sleep Quality Index.

### 3.2. DBAS-16 scores of the sample

The total score of DBAS-16 and the consequences of insomnia subscale score were significant differences among different age, marital status, education level, profession, average working hours per day and average number of night shifts per month. In the two subscales of worry/helplessness and medication, the results of ANOVA showed significant differences among levels of education (worry/helplessness subscale: *F* = 6.382, *p* = 0.002; Drug subscale: *F* = 7.672, *p* = 0.001) and monthly night shifts (worry/helplessness subscale score: *F* = 4.910, *p* = 0.008; Drug subscale score: *F* = 6.223, *p* = 0.002). There were significant differences in the subscale scores of sleep expectation among different genders, ages, marital status, education level, occupation and average night shifts per month (all *p* < 0.05). In addition, the DBAS-16 total score and four subscales score of poor sleepers were higher than those of good sleepers (DBAS-16 total score: *t* = 7.622, *p* = 0.001; consequences subscale score: *t* = 5.801, *p* < 0.001; worry/helplessness subscale score: *t* = 8.357, *p* < 0.001; expectations subscale score: *t* = 5.572, *p* < 0.001 and medication subscale score: *t* = 3.845, *p* < 0.001). The specific results are shown in Supplementary Table S2.

### 3.3. Correlation between PSQI and DBAS-16 sub-scale scores

As shown in Table 2, the total score of PSQI was positively correlated with the total score of DBAS-16 and the scores of four subscales. In addition, subscales of PSQI such as subjective sleep quality and sleep latency were correlated with all DBAS-16 subscales and DBAS-16 total score. Sleep duration was correlated with consequences subscale, worry/helplessness subscale, medication subscale, and total DBAS-16 score. Sleep disturbances, sleeping medication and daytime dysfunction were correlated with DBAS-16 consequences scale, DBAS-16 worry/helpless subscale, DBAS-16 medication subscale and DBAS-16 total score. The specific data of PSQI scores are shown in our previous study (27).

**Table 2:** Correlation between PSQI subscale and DBAS-16 subscale.

Variables	DBAS-16 consequences	DBAS-16 worry/helplessness	DBAS-16 expectations	DBAS-16 medication	DBAS-16 total score
subjective sleep quality	0.242^**^	0.377^**^	0.198^**^	0.105^*^	0.307^**^
sleep latency	0.239^**^	0.391^**^	0.211^**^	0.140^**^	0.322^**^
sleep duration	0.122^*^	0.168^**^	−0.017	0.137^**^	0.144^**^
sleep efficiency	0.058	0.028	0.022	0.024	0.042
sleep disturbances	0.258^**^	0.301^**^	0.211^**^	0.187^**^	0.302^**^
sleeping medication	0.150^**^	0.167^**^	0.105^*^	0.270^**^	0.208^**^
daytime dysfunction	0.296^**^	0.290^**^	0.346^**^	0.132^**^	0.324^**^
PSQI total score	0.330^**^	0.412^**^	0.279^**^	0.206^**^	0.392^**^

^*^p < 0.05, ^**^p < 0.01.

Abbreviations: DBAS-16, Dysfunctional Beliefs and Attitudes about Sleep Scale; PSQI, Pittsburgh Sleep Quality Index.

## 4. Discussion

Our results showed that false beliefs about sleep were prevalent among HCWs, especially in those who were older, married, had a bachelor’s degree or above, were nurses, worked more hours per day, and had more night shifts per month. In addition, we confirmed that there is a positive correlation between DBAS-16 scores and PSQI scores, that is, distorted beliefs and attitude about sleep are associated with poor sleep quality, which is consistent with relevant research results (28, 29). Therefore, the intervention of HCWs’ false beliefs and attitude about sleep contributed to the improvement and recovery of sleep disorders.

First, nearly 77% of HCWs revealed false beliefs about sleep in our study, which is lower than those recently reported by Janati et al. in Morocco for the general population (24). Besides, 25% of HCWs reported sleep disorders, which was lower than reported in previous meta-analyzes and studies in other regions or countries during the COVID-19 pandemic. For example, previous meta-analyzes of insomnia in China indicated that the pooled prevalence of insomnia among HCWs ranged from 35 to 46% (30–32). The pooled prevalence of insomnia symptoms was 28% in Africa and 35% in Latin America (33, 34). An international collaborative study from 13 countries found that the prevalence of insomnia among adults was 37% during the first wave of the pandemic (35). However, prior to the COVID-19 pandemic, the meta-analyzes showed that the prevalence of insomnia was 7.4% in the general population and 18.5% in college students (36, 37), both of which were lower than our findings.

Meta-analyzes indicated women are more likely to experience symptoms of insomnia, anxiety and depression during the COVID-19 pandemic (31, 38, 39). Interestingly, our results suggested that there were no significant differences in sleep beliefs between men and women. The gender difference in insomnia may be due more to a complex interplay of other biological, psychological and social factors (40). Younger people appear to be associated with worse mental health outcomes and more sleep problem (35, 41). However, in this study, we found better sleep beliefs in the younger group. This might be due to the fact that younger HCWs recover more quickly from symptoms of anxiety and depression (42). Liu Y et al. found that married HCWs had a high risk of mental symptoms (43). Similarly, we found that married HCWs had higher DBAS-16 scores than unmarried HCWs. The main reason might be related to their higher family burdens. Our study also found that HCWs with higher level education had more false sleep beliefs, the main reason may be that they were more prone to anxiety and depression and suffered additional pressure from scientific research and work (44). Moreover, our results showed that nurses’ DBAS-16 scores were higher than those medical technicians and administrative staff. In fact, nurses suffered more often from sleep problems and symptoms of anxiety and depression than doctors during the COVID-19 pandemic (45). The main reason might be that nurses needed to spend longer time in contact with patients, and they were faced with a higher risk of infection.

In terms of working hours, our results showed that the participants with longer working hours and more night shifts had higher DBAS-16 scores. On the one hand, it might be because the extra work time brought a greater workload, and they sleep less time. More night duty was the risk factor of developing depressive and anxiety symptoms (46). On the other hand, artificial light at night interferes with the circadian rhythm. Circadian dysregulation impairs cognitive function, increases the risk of severe sleepiness, and causes errors of attention. Additionally, circadian misalignment is a specific risk factor for viral diseases, including the COVID-19 disease (47). Therefore, it is necessary to have a flexible working schedule that is appropriate to the current situation so that HCWs can get adequate rest.

Finally, we compared poor and good sleepers and found that poor sleepers had higher DBAS-16 scores, which was consistent with the results of previous studies (10, 28, 48), suggesting that cognitive factors might increase the risk of developed sleep disorders. The four subscale scores of DBAS-16 were all correlated with the total score of PSQI, among which the worry/helplessness subscale had the highest correlation with the PSQI total score, which means that feelings of worry may play a leading role in the effect on sleep quality. Indeed, sleep quality of HCWs was affected by anxiety, depression and other emotions under the COVID-19 epidemic, and false sleep beliefs may affect sleep and lead to the maintenance and development of insomnia (49).

Though the study of health care workers’ sleep cognition during COVID-19 yielded a lot of useful data, there were some limitations. The study used a cross-sectional design and limited our ability to establish exact causality, and we call for more cohort studies to examine the effect of time. Second, the Yijishan Hospital Affiliated to Wannan Medical College from which the subjects of our study came is the only one designated by Wuhu Municipal government for COVID-19 treatment, so our results may not be applicable to all populations, and we call for future research in other countries or regions.

## 5. Conclusion

The false beliefs about sleep of HCWs should be corrected, and we should provide adequate rest time and psychological support to HCWs with poor sleep.

## Data availability statement

The raw data supporting the conclusions of this article will be made available by the authors, without undue reservation.

## Author contributions

WW: conceptualization, investigation, data curation, and writing - original draft. XJ and H-YG: investigation, data curation, and writing. MT and LJ: investigation and writing - review and editing. MC: investigation, data curation, and writing. HY and HP: conceptualization and writing - review and editing. All authors contributed to the article and approved the submitted version.

## Funding

This study was supported by the National Innovation and Entrepreneurship Training Program for College Students (grant number: 201910368054) and the Young and Middle-aged Research Foundation of Wannan Medical College (grant number: WKS2022F03).

## Conflict of interest

The authors declare that the research was conducted in the absence of any commercial or financial relationships that could be construed as a potential conflict of interest.

## Publisher’s note

All claims expressed in this article are solely those of the authors and do not necessarily represent those of their affiliated organizations, or those of the publisher, the editors and the reviewers. Any product that may be evaluated in this article, or claim that may be made by its manufacturer, is not guaranteed or endorsed by the publisher.
