# Supplementary material for: Investigation on sleep-related cognition of Chinese health care workers during the first wave of COVID-19 pandemic
Source: Front Psychiatry. 2023 Mar 13;14:1019837. doi: 10.3389/fpsyt.2023.1019837 (PMC10040544; doi:10.3389/fpsyt.2023.1019837)
Supplement: Supplementary file 1 [file Table_1.DOCX]

**Supplemental Table 2.** DBAS-16 total score and subscale score in different groups

| Variables | N | Consequences of insomnia | Worry/helpless-ness about sleep | Expectations for sleep | Medication | DBAS-16 total score |
| --- | --- | --- | --- | --- | --- | --- |
| Gender | | | | | | |
| Male | 182 | 5.23±1.93 | 5.37±1.74 | 6.14±2.38 | 3.69±2.07 | 5.11±1.56 |
| Female | 222 | 5.33±1.94 | 5.20±1.90 | 6.63±2.46 | 10.63±6.39 | 5.11±1.72 |
| *t* |  | 0.548 | 0.918 | 2.009 | 0.716 | 0.02 |
| *p*-value |  | 0.584 | 0.359 | 0.045 | 0.474 | 0.984 |
| Age (years) | | | | | | |
| 18~ | 250 | 4.96±1.90^a^ | 5.15±1.72^a^ | 5.90±2.29^a^ | 3.55±2.12 | 4.88±1.61^a^ |
| 24~ | 78 | 5.52±1.91^b^ | 5.27±2.02^ab^ | 6.96±2.21^b^ | 3.55±2.13 | 5.24±1.77^ab^ |
| ≥30 | 76 | 6.12±1.77^c^ | 5.73±1.91^b^ | 7.53±2.25^b^ | 3.88±2.00 | 5.73±1.51^b^ |
| *F* |  | 11.757 | 3.071 | 16.806 | 0.786 | 8.280 |
| *p*-value |  | <0.001 | 0.047 | <0.001 | 0.456 | <0.001 |
| Marital status | | | | | | |
| Unmarried | 322 | 5.14±1.92 | 5.20±1.78 | 6.15±2.38 | 3.60±2.14 | 5.00±1.64 |
| Married | 82 | 5.87±1.89 | 5.59±1.98 | 7.45±2.38 | 3.64±1.94 | 5.54±1.63 |
| *t* |  | 3.080 | 1.747 | 4.402 | 0.140 | 2.682 |
| *p*-value |  | 0.002 | 0.081 | <0.001 | 0.889 | 0.008 |
| Education level | | | | | | |
| Below bachelor | 53 | 4.01±1.74^a^ | 4.46±1.68^a^ | 5.74±2.10^a^ | 2.64±1.95^a^ | 4.14±1.53^a^ |
| Bachelor | 280 | 5.38±1.88^b^ | 5.43±1.81^b^ | 6.27±2.47^a^ | 3.83±2.10^b^ | 5.22±1.64^b^ |
| Master / Doctor | 71 | 5.85±1.89^b^ | 5.31±1.86^b^ | 7.49±2.34^b^ | 3.45±2.00^b^ | 5.40±1.55^b^ |
| *F* |  | 16.150 | 6.382 | 9.851 | 7.672 | 11.490 |
| *p*-value |  | <0.001 | 0.002 | <0.001 | 0.001 | <0.001 |
| Profession | | | | | | |
| Clinician | 184 | 5.40±1.89^ab^ | 5.22±1.78^ab^ | 6.23±2.45^a^ | 3.64±1.95 | 5.11±1.57^ab^ |
| Nurse | 29 | 6.08±2.24^a^ | 6.06±2.50^a^ | 7.79±3.00^b^ | 4.01±2.30 | 5.90±2.18^a^ |
| Medical technician | 108 | 4.97±1.87^b^ | 5.14±1.61^ab^ | 6.50±2.25^a^ | 3.37±2.12 | 4.93±1.53^b^ |
| Administrative staff | 21 | 4.67±1.51^b^ | 4.94±1.70^b^ | 5.79±2.17^a^ | 3.10±1.88 | 5.24±1.80^b^ |
| Other | 62 | 5.36±2.02^ab^ | 5.43±1.94^ab^ | 6.36±2.33^a^ | 3.91±2.44 | 5.11±1.65^ab^ |
| *F* |  | 2.717 | 1.821 | 3.039 | 1.271 | 2.599 |
| *p*-value |  | 0.030 | 0.124 | 0.017 | 0.281 | 0.036 |
| Daily working hours (hours) | | | | | | |
| ≤8 | 322 | 5.18±1.98 | 5.20±1.86 | 6.31±2.46 | 3.55±2.10 | 5.62±1.69 |
| >8 | 82 | 5.70±1.68 | 5.59±1.66 | 6.80±2.30 | 3.86±2.10 | 5.46±1.43 |
| *t* |  | 2.200 | 1.756 | 1.618 | 1.196 | 2.121 |
| *p*-value |  | 0.028 | 0.080 | 0.107 | 0.232 | 0.035 |
| Monthly night shifts (times) | | | | | | |
| 0 | 152 | 5.29±1.92^a^ | 5.32±1.80^a^ | 6.31±2.27^a^ | 3.64±2.23^a^ | 5.12±1.61^a^ |
| 1~ | 195 | 5.04±1.84^a^ | 5.06±1.73^a^ | 6.25±2.43^a^ | 3.35±1.91^a^ | 4.88±1.56^a^ |
| ≥5 | 57 | 6.13±2.06^b^ | 5.91±2.09^b^ | 7.25±2.74^b^ | 4.44±2.17^b^ | 5.87±1.84^b^ |
| *F* |  | 7.211 | 4.910 | 3.974 | 6.223 | 8.178 |
| *p*-value |  | 0.001 | 0.008 | 0.020 | 0.002 | <0.001 |
| PSQI | | | | | | |
| <7 | 303 | 4.98±1.84 | 4.87±1.66 | 6.04±2.35 | 3.38±2.00 | 4.77±1.53 |
| ≥7 | 101 | 6.21±1.90 | 6.50±1.78 | 7.54±2.36 | 4.29±2.24 | 6.13±1.58 |
| *t* |  | 5.801 | 8.357 | 5.572 | 3.845 | 7.622 |
| *p*-value |  | <0.001 | <0.001 | <0.001 | <0.001 | <0.001 |

**Note:** Data is expressed as Mean ± SD. Different letter superscripts (^a^, ^b^, ^c^) indicate statistical differences between groups according to Student-Newman-Keuls (SNK) test (*p*<0.05);

**Abbreviations:** DBAS-16, Dysfunctional Beliefs and Attitudes about Sleep Scale; PSQI, Pittsburgh Sleep Quality Index.
